# Fortified Fermented Rice-Acid Can Regulate the Gut Microbiota in Mice and Improve the Antioxidant Capacity

**DOI:** 10.3390/nu13124219

**Published:** 2021-11-24

**Authors:** Na Liu, Likang Qin, Xiafen Lu, Yuxuan Zhao, Song Miao

**Affiliations:** 1 School of Liquor and Food Engineering, Guizhou University, Guiyang 550025, China; na_liu2021@163.com (N.L.); luxiafen2021@163.com (X.L.); lianshen.zhao@foxmail.com (Y.Z.); 2 Department of Food Chemistry and Technology, Teagasc Food Research Centre, Moorepark, Fermoy, P61 C996 Co. Cork, Ireland

**Keywords:** fortified fermentation 1, *Lactobacillus paracasei* H4-11 2, *Kluyveromyces marxianus* L1-1 3, gut microbiota 4, anti-oxidation 5

## Abstract

The study aimed to explore the effects of fortified fermented rice-acid on the antioxidant capacity of mouse serum and the gut microbiota. Hair characteristics, body mass index, intestinal villus height, intestinal crypt depth, serum antioxidant capacity, and gut microbiota of mice were first measured and the correlation between the antioxidant capacity of mouse serum and the gut microbiota was then explored. The mice in the lactic acid bacteria group (L-group), the mixed bacteria group (LY-group), and the rice soup group (R-group) kept their weight well and had better digestion. The mice in the L-group had the better hair quality (dense), but the hair quality in the R-group and the yeast group (Y-group) was relatively poor (sparse). In addition, the inoculation of *Lactobacillus paracasei* H4-11 (*L. paracasei* H4-11) and *Kluyveromyces marxianus* L1-1 (*K. marxianus* L1-1) increased the villus height/crypt depth of the mice (3.043 ± 0.406) compared to the non-inoculation group (R-group) (2.258 ± 0.248). The inoculation of *L. paracasei* H4-11 and *K. marxianus* L1-1 in fermented rice-acid enhanced the blood antioxidant capacity of mouse serum (glutathione 29.503 ± 6.604 umol/L, malonaldehyde 0.687 ± 0.125 mmol/L, catalase 15.644 ± 4.618 U/mL, superoxide dismutase 2.292 ± 0.201 U/mL). In the gut microbiota of L-group and LY-group, beneficial microorganisms (*Lactobacillus* and *Blautia*) increased, but harmful microorganisms (*Candidatus Arthromitus* and *Erysipelotrichales*) decreased. *L. paracasei* H4-11 and *K. marxianus* L1-1 might have a certain synergistic effect on the improvement in antibacterial function since they reduced harmful microorganisms in the gut microbiota of mice. The study provides the basis for the development of fortified fermented rice-acid products for regulating the gut microbiota and improving the antioxidant capacity.

## 1. Introduction

Probiotics are considered as a potential substitute for antibiotics and a live biotherapeutic agent for improving animal health [1]. The human gut microbiota includes 10 to 100 trillion microorganisms, which are mainly bacteria and vastly outnumber human cells. Among the human gut microbiota, the most common bacteria are *Bacteroidetes* and *Firmicutes* [2]. The gut microbiota affects intestinal functions, such as intestinal metabolism and integrity. Different dietary structures can affect the weight difference and metabolism regulation directly and change the gut microbiota [3]. Healthy gut microbiota is characterized by predominant beneficial microbes, whereas dysfunctional gut microbiota is characterized by the predominance of harmful microbes. Interestingly, probiotics in gut microbiota are found in food fermentation processes and also used as over-the-counter supplements because of their proven beneficial effects on the host [4]. Notably, lactic acid bacteria also play a probiotic role in regulating the nutritional status of the body, improving the physiological function of the body, avoiding cell infection, improving the efficacy of drugs, alleviating the effects of toxic substances on the body, promoting immune response, preventing tumorigenesis, and slowing aging [5,6,7,8,9,10]. The role of probiotics in human gut microbiota is widely concerned [2,3,5].

Oxidation processes exist in foods and human metabolism and the antioxidant capacity is a hot topic in the food industry. Antioxidants can inhibit oxidation processes and prevent oxidative stress-related diseases [11] and the physiological effects of antioxidants on human health/organism have been widely explored in recent studies. Some *Lactobacilli* exhibited excellent antioxidant capacity and probiotic function, including *Lactiplantibacillus plantatum* [12], *Lactobacillus casei* [13], *Lactobacillus fermentum* [14], and *Lactobacillus rhamnosus* [15]. Notably, the decrease in oxidative products or oxidase activity and the increase in antioxidant enzyme activity are therapeutically beneficial to the recovery of intestinal injury.

Fortified fermentation technology is widely applied in the food industry. Rice-acid is popular among the consumers in Guizhou Province because of its unique flavor. In recent years, the development of fermented rice and its beneficial effects on the prevention of some diseases, such as cardiovascular disease, cancer, and Alzheimer’s disease, have been extensively explored [16,17,18]. Rice-acid is a fermented rice food. In our previous study [19,20], the fortified fermentation technology improved the flavor of rice-acid. However, the probiotic function of rice-acid and its influence on the gut microbiota remain to be studied.

The study aims to explore the effect of fortified fermented rice-acid on the tissue characteristics, antioxidant capacity, and gut microbiota of mice. We first analyzed the weight and relative organ weight of the mice fed with fermented rice-acid in different experimental groups, then measured the hair characteristics, intestinal villus height, intestinal crypt depth, serum antioxidant capacity, and gut microbiota, and finally explored the correlation between antioxidant capacity and gut microbiota.

## 2. Materials and Methods

### 2.1. Sample Preparation

Fermented rice-acid, rice soup, and saline were first prepared. In the control group (W-group), the mice were fed with 0.85% sterile saline solution. In the lactic acid bacteria group (L-group), the mice were fed with fermented rice-acid inoculated with *Lactobacillus paracasei* H4-11 (*L. paracasei* H4-11) (8.0% selenium rice flour and 7.80 × 10^7^ CFU/mL *L. paracasei* H4-11 for 96-h fermentation at 30 °C). In the yeast group (Y-group), the mice were fed with fermented rice-acid inoculated with *Kluyveromyces marxianus* L1-1 (*K. marxianus* L1-1) (8.0% selenium rice flour and 4.95 × 10^5^ CFU/mL *K. marxianus* L1-1 for 96-h fermentation at 30 °C). In the mixed fermented rice-acid group (LY-group), the mice were fed with fermented rice-acid inoculated with *L. paracasei* H4-11 and *K. marxianus* L1-1 (8.0% selenium rice flour, 7.80 × 10^7^ CFU/mL *L. paracasei* H4-11 and 4.95 × 10^5^ CFU/mL *K. marxianus* L1-1 for 96-h fermentation at 30 °C). In the rice soup group (R-group), the mice were fed with rice soup (prepared with 8% selenium rice flour and boiling 92% water). Rice-acid was prepared with the previous method [19]. Briefly, *L. paracasei* H4–11 (CCTCC 2021074) and *K. marxianus* L1–1 (CCTCC 2021073) were isolated from fermented rice-acid in our previous study [19]. First, the strains *L. paracasei* H4–11 and *K. marxianus* L1–1 were, respectively, activated and cultured in the MRS medium (37 °C for 24 h) and YPD medium (30 °C for 48–72 h) and the cultivation was stopped when the two strains entered the logarithmic phase. Then, the strains were washed with 0.85% sterile saline and the resulting suspensions were used to inoculate fermented rice acid.

### 2.2. Experimental Animals in Various Groups

**Figure 1:**
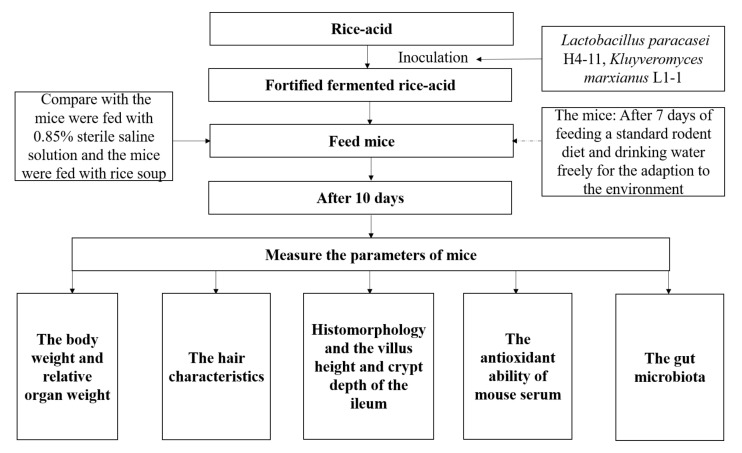
Schematic diagram indicating fortified fermented rice-acid, 0.85% sterile saline solution and rice soup intervention of the mice. Each group took the fortified fermented rice-acid, 0.85% sterile saline solution or rice soup for ten days, respectively.

Sixty 8-week-old SPF male mice with body weight of 23 ± 2 g were purchased from Chongqing Tengxin Biological Co., Ltd. (Chongqing, China) and the laboratory animal production license number was: SCXK (Jing) 2019-0008. The experimental mice were raised in accordance with Regulations for Protection and Use of Experimental Animals of Guizhou University. All experimental procedures were performed according to the Laboratory Animal Welfare Standards and approved by the Subcommittee of Experimental Animal Ethics, Guizhou University (No. EAE-GZU-2021-P005). The schematic diagram of this study is shown in Figure 1. Six mice per cage were free to eat and drink under the following conditions: room temperature of 23 ± 2 °C, relative humidity of 63% ± 5%, and the light–dark cycle (12 h–12 h). After feeding with a standard rodent diet (SPF maintenance feed, the production license number: Beijing Feed Certificate SCXK (Jing) 2019-0008 and drinking water freely (the weight of mouse was 28.04 ± 1.03 g) for 7 consecutive days, the mice were randomly divided into 5 groups (each group containing 12 mice in two cages (Cage 1 and Cage 2)): W-group, L-group, Y-group, LY-group, and R-group. During the experiment, the mice ate and drank freely and were gavaged with 1 mL of corresponding inoculation suspension according to the grouping results in Section 2.1 at a fixed time (9:00 am) every day. Different indexes of the mice in Cage 1 (*n* = 6) were measured and the growth state of the mice in Cage 2 was observed.

### 2.3. Preparation of Serum Samples

After being fed with experimental diets for 10 consecutive days, all the mice fasted and drank freely for 12 h. The mice were sacrificed at 12 h after the last observation. After weighing, blood samples were collected from the orbital sinus by removing the eyeballs under deep anesthesia. Then, collected blood samples were centrifuged at 4 °C and 40,000× *g* for 10 min to prepare serum samples. Finally, the prepared serum samples were stored in an ultra-low temperature freezer at −78 °C for testing.

### 2.4. Determination of Body Mass and Relative Organ Weight of Mice

After measuring the body mass of the mice, the mice were quickly dissected on ice. Heart, liver, spleen, kidney, and small intestine of mice were taken out. After absorbing floating blood with a cotton swab, the organs were weighed and washed with ice-cooled normal saline and residual water was adsorbed with dry filter paper. The relative organ weight was calculated as:

𝐴 (mg/g) = 𝑀1/𝑀2,(1)

where *A* is the relative organ weight; *M*1 is the organ mass (mg); *M*2 is the mouse body mass (g).

### 2.5. Body Hair Characteristics of Mice

After the mice were dissected, the hair samples of the mice in each group were collected. Five hairs from each site of each mouse were examined. The morphological observation was carried out with the aid of a scanning electron microscope (SEM) in order to detect the differences in the morphology and density (the degree of dispersion of hair) of the hair of the mice in each group. Hair stands were withdrawn from mice using scissors, SEM analyses were performed as described previously [21] with JEOL JCM-6000 microscope (Tokyo, Japan) operating at an acceleration voltage of 15 kV. For semi-quantitative assessment of the mouse hair dysmorphology, the previously proposed scale of hair morphological changes was used [21]. In addition, the hair surface microstructure was identified by analyzing the formation of epidermal scales in SEM images [22].

### 2.6. Intestinal Histology and Morphological Analysis

After the mice were euthanized, the entire small intestine was taken out and measured and ileum of small intestine was taken for histological analysis. The intestines were flushed with precooled phosphate buffer saline to remove intestinal contents, and the ileum tissue samples were placed in 10% formaldehyde buffer for 24 h and then embedded in paraffin. The embedded sample (4 μm) was mounted on a glass slide and used with hematoxylin and eosin (HE). In HE-stained glass slides, the altered structure of the mucosa and polymorphonuclear cell infiltration were analyzed with a histopathological grading system according to the previous method [23]. In Image-pro plus 6.0 (Media Cybernetics, Inc., Rockville, MD, USA), 3 fields of view (100×) were selected for each slice in each group to take photos. When photos were taken, the fields of view full of organs were selected to ensure consistent background light in each photo. With the 100× ruler in Image-Pro Plus 6.0 software as the standard, five complete villi in each slice of each sample were selected to measure the villus height (mm), crypt depth (mm), and villus height/crypt depth and calculate their own average values.

### 2.7. Determination of Antioxidant Index

To measure antioxidant enzyme activities, the blood samples were then centrifuged at 4000× *g* and 4 °C for 10 min. The serum supernatant was immediately used to detect enzyme activities. The contents of glutathione (GSH), catalase (CAT), and superoxide dismutase (SOD) in serum were detected according to the instructions of GSH, CAT and T-SOD test kits (Nanjing Jiancheng Bioengineering Institute, Nanjing, China). Briefly, 0.05 mL, 0.1 mL, and 0.05 mL of serum was used in the determination of GSH, CAT, and SOD, respectively. In the reaction between reduced GSH and dithiodinitrobenzoic acid, a yellow compound was formed and the content of reduced GSH could be measured at 405 nm. One unit of CAT was defined as the quantity of enzyme required for decomposing 1 μmol H_2_O_2_ monitored at 405 nm. The SOD activity was detected with a Total Superoxide Dismutase (T-SOD) Assay Kit (Hydroxylamine method). One unit of SOD activity was defined as the quantity of SOD when the inhibition rate of SOD reached 50% per gram tissue in 1 mL of reacted solution and the SOD activity was monitored at 550 nm. The content of malonaldehyde (MDA) in serum was detected according to the instructions of an MDA test kit (Solarbio Life Sciences, Beijing, China). Briefly, serum (0.1 mL) was used for the determination of MDA. Under acidic and high-temperature conditions, MDA was condensed with thiobarbituric acid to produce brown–red 3,5,5-trimethyloxazole-2,4-dione, whose maximum absorption wavelength is at 532 nm. Soluble sugars interfered with the absorption of MDA at 450 nm and 600 nm. Therefore, the difference in absorbance at 532 nm, 450 nm and 600 nm could be used to calculate the content of MDA.

### 2.8. Determination of the Gut Microbiota

After the mice were dissected, the cecal contents were aseptically removed and the collected products were transferred into a 2 mL cryotube, which was quickly placed in a liquid nitrogen tank and then stored at −80 °C for later use. Three replicates were arranged randomly for each group in the same cage and a total of 15 samples were transported to Beijing Nuohe Zhiyuan Technology Co., Ltd. (Beijing, China). Through the amplification of the full length of 16S, the construction of an SMRT Bell library, and sequencing with the PacBio platform (Pacific Biosciences, Menlo Park, CA, USA), based on bioinformatics, the microbial diversity and community composition differences in the small intestine of mice were analyzed [24].

#### 2.8.1. Genomic DNA Extraction and Amplicon Generation

Total genome DNA was extracted from samples with the Cetyltrimethylammonium Bromide and sodium dodecyl sulfate (CTAB/SDS) method. DNA concentration and purity were monitored on 1% agarose gels. According to the monitored concentration, DNA was diluted to 1 ng/μL with sterile water. The 16S rRNA genes of distinct regions were amplified by using specific primer with the barcode. All PCR reactions were carried out with Phusion® High-Fidelity PCR Master Mix with GC Buffer (New England Biolabs, Beverly, MA, USA).

#### 2.8.2. Mixing and Purification of PCR Products

The same volume of 1X loading buffer (contained SYB green) was mixed with PCR products to carry out electrophoresis on 2% agarose gel for detection. PCR products were mixed in equidensity ratios. Then, mixed PCR products were purified with a QIAquick@ Gel Extraction Kit (QIAGEN, Hilden, Germany).

#### 2.8.3. Library Construction and Sequencing

With DNA binding enzyme, sequencing adapters were connected to both ends of the amplified DNA fragments, which were purified with AMpure PB magnetic beads to construct a SMRT Bell library. After the purified fragments were re-dissolved in the buffer, the BluePipin fragments were used to screen the fragments of a specific size and the DNA fragments were purified by AMpure PB magnetic beads (Pacific Biosciences, Menlo Park, CA, USA). The constructed library was quantified based on Qubit concentration and the insert fragments were detected by Agilent 2100 and then sequenced on the PacBio platform (Pacific Biosciences, Menlo Park, CA, USA).

OTUs with 97% similarity were used for alpha diversity estimations, including observed species, community diversity (Shannon, Simpson), community richness (Chao, Abundance-based Coverage Estimator (ACE)), and sequencing depth (the Good’s coverage) and PD_whole_tree. All these indices in our samples were calculated with Quantitative Insights Into Microbial Ecology (QIIME) software (v1.9.1) (Rob Knight team, University of California, San Diego, USA) and displayed with R software (v2.15.3) (MathSoft, Cambridge, MA, USA). To display the differences among samples, Beta diversity was calculated with the Unweighted Pair-group Method with Arithmetic Means (UPGMA) Clustering to interpret the distance matrix in QIIME software (v1.9.1) (Rob Knight team, University of California, San Diego, USA). Non-Metric Multi-Dimensional Scaling (NMDS), the distance between points, can reflect the differences between groups and within groups of samples. Linear discriminant analysis (LDA) Effect Size (LEfSe) analysis can search for biomarkers with statistical differences among groups. The LDA value distribution histogram shows the species with LDA Score greater than the set value, 4.

### 2.9. Statistical Analysis

Data were reported as the mean ± standard deviation (SD). The statistical significance between groups was evaluated by one-way ANOVA, followed by Waller–Duncan multiple range test in SPSS Statistics 20.0 (IBM, New York, USA). *p* < 0.05 was considered to be statistically significant. The indices in gut microbiota were calculated with QIIME software (v1.9.1) and displayed with R software (v2.15.3). In order to minimize the influence of individual differences of mice on the overall evaluation results, we used Spearman’s correlation coefficient [25,26,27] to analyze the correlations between the key microbial genera and other parameters, and the heatmaps were made using ORIGIN PRO 2018 (OriginLab Corporation, Northampton, MA, USA).

## 3. Results and Discussion

### 3.1. Analysis of Mouse Body Weight and Relative Organ Weight

After the treatment, the weight of mice was heavier (35.10~38.81 g) than that of the mice adapted to the environment for 7 days (28.04 ± 1.03 g). Compared with the weight of control group (38.63 ± 2.07 g), the mice in the other four groups of experiments were lighter, but the difference between Y-group and W-group was not significant (Table 1). The relative heart weight of L-group was larger than that of W-group, but the relative heart weights of the other three groups were smaller than that of W-group (*p* < 0.05). The relative liver weight of Y-group was smaller than that of W-group and the relative liver weights of other three groups were all greater than that of W-group (*p* < 0.05). The relative kidney weights of mice in the other four groups of experiments were lower than that of W-group. The relative small intestine weights of the four experimental groups were greater than that of W-group.

Except for Y-group, the other experimental groups showed no significant difference in the relative spleen weight compared to W-group because a spleen is an essential organ related to anti-inflammatory reflex and immune function [28]. The result indicated that the mice in L-group and LY-group had the similar immune functions with W-group. Relative liver weight refers to the ratio of liver wet weight to body weight. It indicates the health status of the liver and is related to obesity [29]. The relative liver weight of the mice in Y-group was close to that of W-group, indicating that the mice in the Y-group might have the similar absorption capacity with W-group. The mice in L-group, LY-group, and R-group could keep their weight well, indicating that the mice in the three groups had the higher digestion capacity than others. The body weight gain of the mice is dependent on the caloric density of food, total energy intake, and activities, so the weight loss effect observed in these groups will be further explored. The function related to the relative organ weight in Section 3.1 needs to be further studied in subsequent experiments.

### 3.2. Analysis of Mouse Hair Characteristics

**Figure 2:**
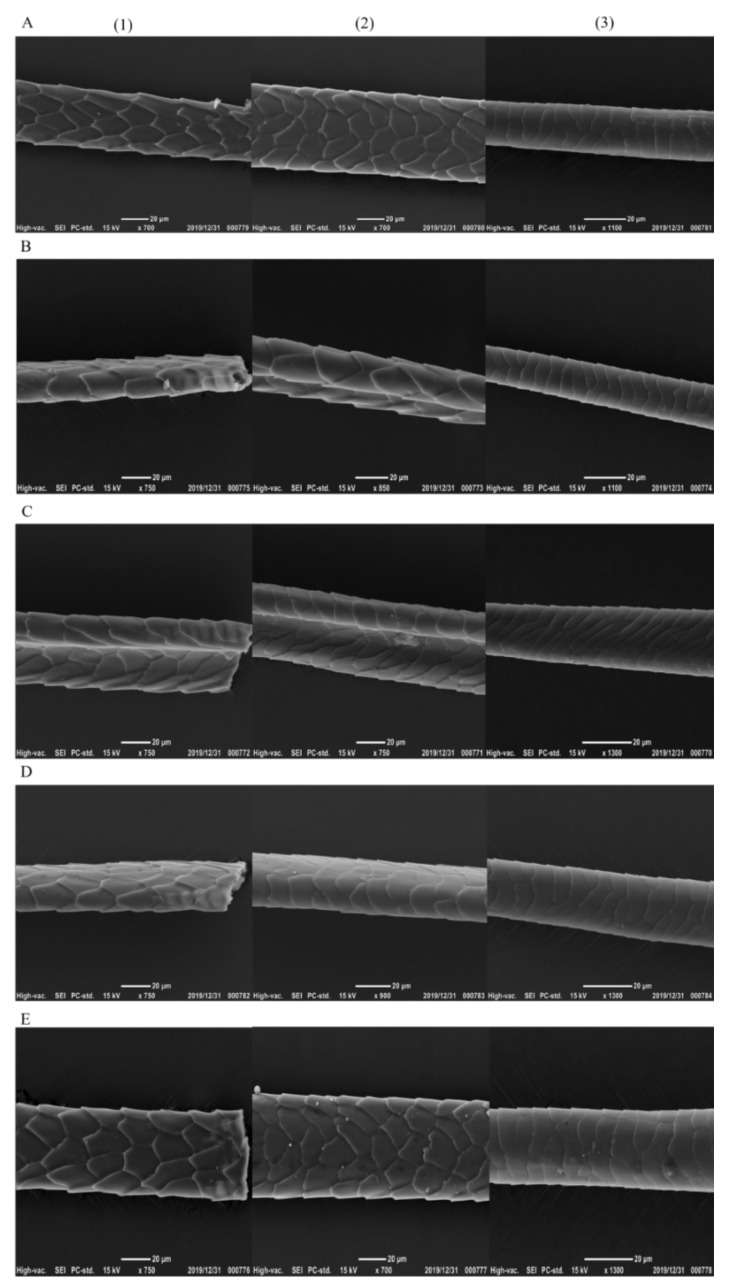
The hair characteristics of mice (**A**–**E** refer to L-group, Y-group, LY-group, R-group and W-group, respectively, (**1**)–(**3**) refer to the head, middle and tail of the mice tail, respectively).

The hair quality is correlated with physical health or diseases [30]. Compared with the hair of the mice in the control group, the hair in the other four groups did not show dysmorphology, and the mice in the other four groups had the regular, contact, and staggered hair scales with shiny surfaces (Figure 2). The scales on the head, middle, and tail of mouse hair of each group were staggered. The head part of mouse hair in L-group and W-group was denser than that in LY-group, R-group and Y-group, and the head part of mouse hair in L-group had sharp ends or bifurcations. The hair density in the middle part of mice was higher than that in the head part. The hair density in the middle part of mice in L-group and W-group was still higher, followed by LY-group, and R-group and Y-group. The hair density in the tail part of mice showed no significant difference among various groups and the hair density in the tail part of mice was lower than that in the middle part. The result suggested that the mice in L-group might have the high-quality hair characteristics (dense), whereas those in R-group and Y-group had the poor hair quality (sparse). The difference will be further explored in the future.

### 3.3. Analysis of Histomorphology and the Villus Height and Crypt Depth of the Ileum in Mice

**Figure 3:**
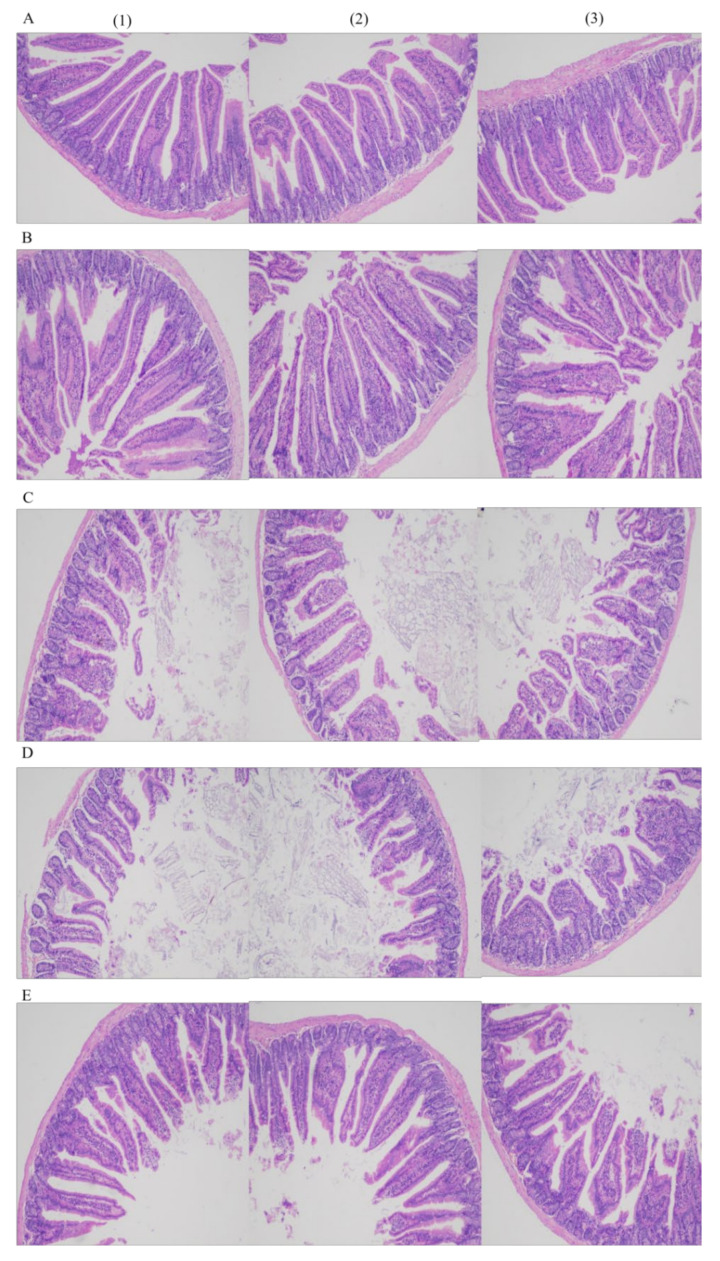
The morphology of terminal ileum (100×) in each group (**A**–**E** refer to L-group, Y-group, LY-group, R-group and W-group, respectively, (**1**)–(**3**) refer to three 100× field of view).

A crypt is considered as the villus factory. Deeper crypts indicate fast tissue turnover to permit the renewal of villi in response to normal sloughing or inflammation caused by pathogens or their toxins [31]. The morphology of the mouse ileum is shown in Figure 3. The mice in all the groups showed normal histological morphology of the ileum without edema, inflammatory cell infiltration, and other pathological changes.

Villus height and crypt depth in the five groups were measured (Table 2). Villus heights in various groups decreased according to the following order: Y-group > L-group > W-group > LY-group > R-group. Crypt depths in various groups increased according to the following order: LY-group < W-group < R-group < Y-group < L-group. Higher villus height and shallower crypt depth indicate the higher nutrient absorption capacity of mice. Importantly, the ratio of villus height to crypt depth (villus height/crypt depth, V/C) is positively correlated with the growth rate of mice and crypt depth indicates the activity and function of intestinal stem cells [32]. Villus height and crypt depth of intestine are important indicators to measure the digestion and absorption capacity of intestine. The villus height/crypt depth reflects the functional status of the mice [32]. The decrease in the V/C indicates the damaged mucosa and the decreased digestion and absorption capacity. The V/C in W-group (3.270 ± 0.591) was significantly lower than that in Y-group (3.934 ± 0.681), indicating that the digestion capacity and absorption capacity were higher in the mice in Y-group. However, the V/C of R-group (2.258 ± 0.248) was significantly reduced, indicating that the digestion and absorption capacity was weak in the mice in R-group. The V/C in L-group (3.165 ± 0.405) was not significantly different from that in LY-group (3.043 ± 0.406) and the data of the two groups were different from that in W-group. This indicator showed that lactic acid bacteria-enhanced fermentation of rice-acid had a small effect on the digestion and absorption capacity of mice. Cucick et al. [33] also found that the ratio of villus height to crypt depth in the mice fed with milk fortified by lactic acid bacteria was similar to that of mice fed with milk containing commercial folic acid. In addition, Carrizo et al. confirmed that Quinoa pasta fermented with lactic acid bacteria could increase villus height of mice, but it had little effect on crypt depth [34]. The above results showed that rice-acid fermented with lactic acid bacteria could increase the V/C of mice.

### 3.4. Analysis of the Antioxidant Ability of Mouse Serum

The analysis results of antioxidant capacity of mouse serum are shown in Table 3. GSH is a low-molecular-weight antioxidant and acts as an important antioxidant enzyme that catalyzes the reduction of peroxide. It can maintain cell redox balance, prevent oxidative damage, and remove O_2_^⋅−^, ^⋅^OH, and H_2_O_2_. GSH content is an important factor to indicate the body’s antioxidant capacity [35]. The GSH content in W-group was lower than that in the other groups. The GSH content in Y-group was the highest (36.735 ± 4.515 μmol/L), followed by LY-group. GSH content showed no significant difference between L-group and R-group (*p* > 0.05).

MDA is a product of lipid peroxidation caused by free radicals in the body and often used as an indicator to evaluate the oxidation level [36]. MDA contents in various groups decreased according to the following order: R-group > W-group > Y-group > L-group > LY-group. The MDA contents in R-group and W-group were higher than that in L-group and LY-group (*p* < 0.05) and the MDA content in LY-group was the lowest (0.687 ± 0.125 mmol/L). The MDA content showed no significant difference between LY-group and L-group (*p* > 0.05). The difference in MDA content between R-group and W-group was not significant (*p* > 0.05).

CAT is an enzyme that catalyzes the decomposition of H_2_O_2_ into oxygen and water and exists in all tissues of all known animals [36]. CAT content in LY-group (15.644 ± 4.618 U/mL) was significantly higher than that in W-group (*p* < 0.05) and CAT content in L-group (11.624 ± 3.111 U/mL) was higher than that in W-group. CAT contents in Y-group and R-group were lower than that in W-group. The difference in CAT content between Y-group and R-group was not significant (*p* > 0.05).

SOD can catalyze the dismutation of superoxide and act as a superoxide radical scavenger in an organism. It can convert harmful superoxide free radicals into H_2_O_2_ and CAT and peroxidase oxidase in the body decompose the generated H_2_O_2_ into water. As an antioxidant enzyme, SOD can effectively remove superoxide anion radicals in the body and reduce the production of MDA and metabolites of free radicals [37], thus protecting the body from cytotoxic damage. SOD contents in various groups decreased in the following order: LY-group > L-group > R-group > Y-group > W-group. The differences in SOD content between the two groups (LY-group and L-group) and other groups (Y-group, R-group and W-group) were significant (*p* < 0.05). LY-group had the highest SOD content (2.292 ± 0.201 U/mL).

The above data indicated that fermented rice-acid could enhance the antioxidant capacity of the body. Especially, LY-group performed best, followed by L-group. The difference in antioxidant capacity between LY-group and L-group was not significant, indicating that rice-acid fermented with *L. paracasei* H4-11 could reduce the content of free radicals in the body and effectively protect important tissues from damage. In summary, fortified fermented rice-acid had the good antioxidant capacity and its mechanism might be related to the composition of gut microbiota. Lun et al. found that the improvement in the gut microbiota of mice treated with tea polyphenols was closely related to the enhancement of antioxidant ability [38]. Antioxidants contribute to human health through preventing aging, inflammation, infection, and many diseases (including cancers, cataracts, diabetes, and neurodegenerative diseases) [11,39,40]. The results provide the basis for the further development and utilization of probiotic properties of rice-acid. In this study, we analyzed the serum content of antioxidants. Some researchers analyzed the antioxidants of serum with SOD, CAT, GSH and other parameters [41,42,43]. In our future study, we will focus on the antioxidants of erythrocyte and intestinal tissue.

### 3.5. Analysis of the Gut Microbiota in Mice

#### 3.5.1. Analysis of the Microbial Diversity in Small Intestine of Mice

**Figure 4:**
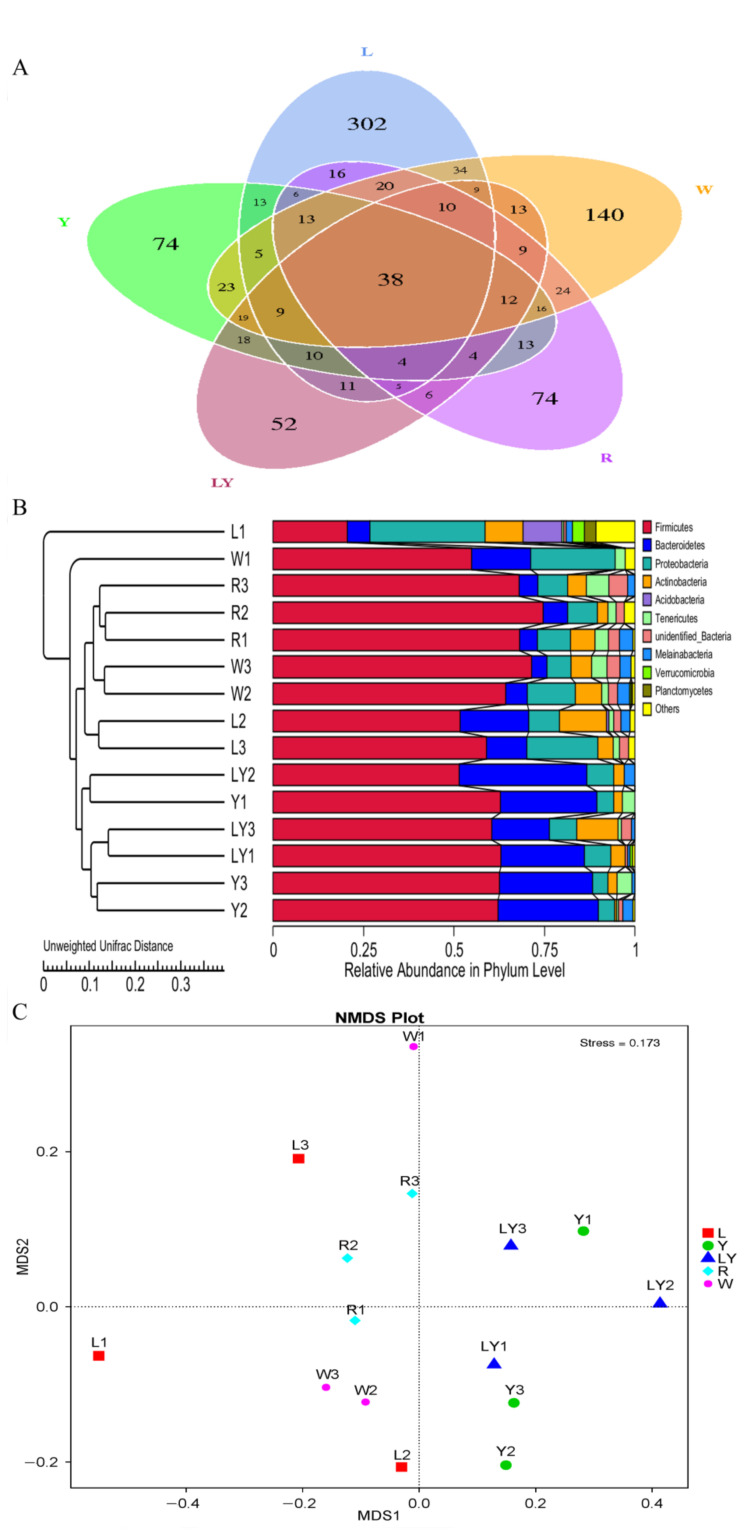
Species distribution of intestinal flora of mice in different experimental groups (L-group, Y-group, LY-group, R-group and W-group) (**A**) Venn diagram, (**B**) Unweighted Pair-group Method with Arithmetic Means (UPGMA) clustering tree based on Unweighted Unifrac distance, (**C**) Non-Metric Multi-Dimensional Scaling (NMDS) analysis (MDS1 is the abbreviation of Multi-Dimensional Scaling 1 and MDS2 is the abbreviation of Multi-Dimensional Scaling 2).

The alpha diversity was selected to evaluate different types of gut bacteria in five groups [44]. Table 4 shows sequencing analysis results of gut microbiota of the mice in five groups. The number of observed species ranged from 108 in LY-group to 202 in L-group and the different sequencing data were related to the different experimental designs in five groups. Chao index ranged from 148.113 (LY-group) to 287.020 (W-group) and Ace index ranged from 159.118 (LY-group) to 321.170 (W-group). Two indexes indicated that the intestinal floral abundances in five groups were different. The Shannon index ranged from 2.674 (LY-group) to 3.582 (L-group) and the Simpson index ranged from 0.566 (W-group) to 0.781 (Y-group). Two indexes indicated that L-group and Y-group had the richer diversity of gut microbiota. The good’s coverage ranged from 0.977 to 0.990, indicating that sequencing depth met experimental requirements. PD_whole_tree ranged from 13.465 (LY-group) to 24.576 (L-group), indicating that the phylogenetic diversity of L-group was the most abundant, whereas the phylogenetic diversity of LY-group was low. A Venn diagram (Figure 4A) shows that the total number of common OTUs in five groups was 38. The number of common OTUs indicated that microbial species in the gut of mice in different groups were partially the same. The numbers of unique OTUs in L-group, Y-group, LY-group, R-group, and W-group were, respectively, 302, 74, 52, 74, and 140. The differences in intestinal microbial species of the mice under different treatments further interpreted the diversity of gut microbiota.

The β-diversity analysis was performed in order to evaluate the difference in the diversity of gut flora [44]. UniFrac distance and NMDS (Figure 4B,C) indicated a complete separation of the microbial community composition between W-group and L-group/LY-group. In addition, the microbial community composition of LY-group was relatively close to that of L-group and Y-group. The results indicated that both rice-acid fermented with *L. paracasei* H4-11 and *K. marxianus* L1-1 and rice-acid fermented with single starter could change the composition of gut microbiota of mice.

#### 3.5.2. Distribution of Dominant Microbial Species in the Small Intestine of Mice

**Figure 5:**
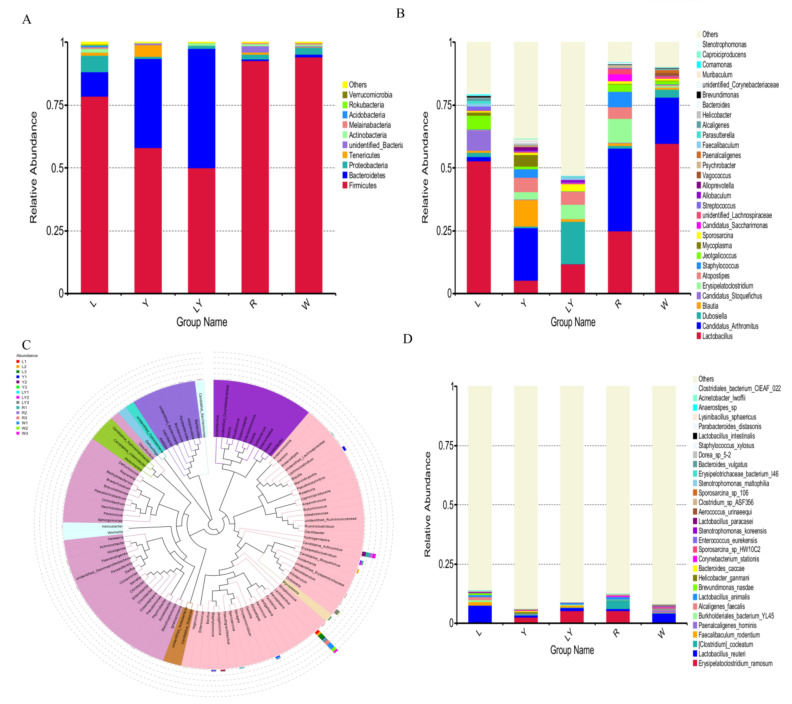
Microbial distribution of mice’s intestinal flora in different experimental groups (L-group, Y-group, LY-group, R-group and W-group): (**A**) phylum level, (**B**) genus level, (**C**) genus level evolutionary tree, (**D**) species level.

Intestinal microorganisms have a crucial influence on the intestinal microenvironment. Among the top 10 phyla in five groups, the dominant phyla were Firmicutes and Bacteroidetes (Figure 5A). In particular, the relative abundances of Firmicutes in R-group and W-group were larger and, respectively, reached 92.78% and 94.20%. The relative abundances of Bacteroidetes in LY-group, Y-group and L-group were, respectively, 47.66%, 35.37%, and 9.65%. The phyla of Firmicutes (Gram-positive low G + C mol% branch) and Bacteroidetes (Gram-negative bacteria) as probiotics were the dominant phyla in the gut microbiota [45]. In addition, the increase in the abundance ratio of Firmicutes to Bacteroidetes in the intestine could make the mice absorb energy more efficiently and lead to obesity [46,47]. In this study, the abundance ratios of Firmicutes to Bacteroidetes in various groups decreased in the following order: R-group > W-group > L-group > Y-group > LY-group, indicating that rice-acid fermented with the two strains (*L. paracasei* H4-11 and *K. marxianus* L1-1) as the feed of mice might effectively reduce the weight of mice. The result needs to be further explored in subsequent experiments.

Figure 5B shows the top 30 genera with a high relative abundance. The microbial genera in various groups were significantly different. Top 10 genera included *Lactobacillus*, *Candidatus Arthromitus*, *Dubosiella, Blautia*, *Candidatus Stoquefichus*, *Erysipelatoclostridium*, *Atopostipes*, *Staphylococcus*, *Jeotgalicoccus,* and *Mycoplasma*. Among them, *Lactobacillus* and *Candidatus Arthromitus* were the most abundant genera among five groups. The relative abundances of *Lactobacillus* in various groups decreased in the following order: W-group (59.80%) > L-group (52.86%) > R-group (24.97%) > LY-group (11.80%) > Y-group (5.38%). The difference in the abundance of *Lactobacillus* between L-group and W-group was not significant, but the abundance of *Lactobacillus* in L-group and W-group was significantly higher than that in R-group (*p* < 0.05). Some strains of *Lactobacillus* survive in the gut of human and animals and reach gastrointestinal tracts [48]. *Lactobacillus* has a certain antibacterial effect and can control the shelf life of foods and shorten the fermentation period [48]. *Lactobacillus* induces the expression of anti-inflammatory genes, improves gut function and motility, and modulates immune response [49]. The relative abundances of *Blautia* in Y-group, LY-group, and Y-group were higher than that in W-group. *Blautia* was significantly reduced in patients with colorectal cancer and could prevent inflammation, promote the production of short chain fatty acids, and maintain intestinal homeostasis activity, displaying probiotic properties [50]. The relative abundances of *Candidatus Arthromitus* in various groups decreased in the following order: R-group (32.95%) > Y-group (20.91%) > W-group (18.28%) > L-group (1.60%) > LY-group (<0.01%). The relative abundances of *Candidatus Arthromitus* in LY-group and L-group were significantly lower than that in W-group. *Candidatus Arthromitus* may be a kind of microorganism indicating the state between health and disease and many functions of *Candidatus Arthromitus* have not been studied [50]. The result indicated that *L. paracasei* H4-11 could inhibit the growth of *Candidatus Arthromitus*. It was further speculated that the compositions of gut microbiota in the mice in LY-group and L-group were healthier. The phylogenetic relationships of the representative sequences of the top 100 genera were obtained through multiple sequence alignments (Figure 5C). The 100 genera belonged to 10 different phyla. *Lactobacillus* was detected in all 15 samples in the five experimental groups and had the largest relative abundance. *Candidatus Arthromitus* was detected in 14 samples (except LY2). *Dubosiella*, which belongs to the family *Erysipelotrichaceae* and is related to *Allobaculum stercoricanis* [51], was detected in 15 samples, and the relative abundances of *Dubosiella* in L-group and Y-group were lower than that in W-group. The relative abundance of *Allobaculum spp*. was increased in the animal study on the diet of glycated or advanced glycation end products [52]. *Erysipelatoclostridium* was detected in all the groups except L-group. An increase in *Erysipelatoclostridium* led to an increase in pathogenic bacteria in patients with gout [53]. This result suggested that L-group might prevent the production of pathogenic bacteria to a certain degree. Similarly, previous findings demonstrated that *Erysipelotrichales* was enriched in the control diet group other than the advanced glycation end product diet group [54,55]. The phylogenetic relationships among these genera further confirmed the diversity and distribution of gut microbiota in mice of different groups.

Figure 5D shows the top 30 species with a high relative abundance. The microbial species showed significant differences among five experimental groups. The top 10 species with a high abundance were *Erysipelatoclostridium*, *Lactobacillus reuteri*, *(Clostridium) cocleatum*, *Faecalibaculum rodentium*, *Paenalcaligenes hominis*, *Burkholderiales bacterium*, *Alcaligenes faecalis*, *Lactobacillus animalis*, *Brevundimonas nasdae*, and *Helicobacter ganmani*. *Erysipelatoclostridium* and *Lactobacillus reuteri* were the two dominant species. *L. paracasei* H4-11 was detected in L-group, Y-group, and LY-group, but *L. paracasei* H4-11 was not detected in R-group or W-group, indicating that *L. paracasei* H4-11 in rice-acid could be well absorbed and utilized by mice. In the mice in Y-group fed with rice-acid fermented without *L. paracasei* H4-11, *L. paracasei* H4-11 was detected in small intestine of mice, indicating that *K. marxianus* L1-1 in Y-group contributed to the growth of *L. paracasei*. It was preliminarily speculated that the two strains (*L. paracasei* H4-11 and *K. marxianus* L1-1) might be related to antioxidant capacity. According to previous reports [38,56], antioxidant capacity was related to the change in gut microbiota.

#### 3.5.3. Microbial Species with Significant Abundance Difference in Small Intestine of Mice

**Figure 6:**
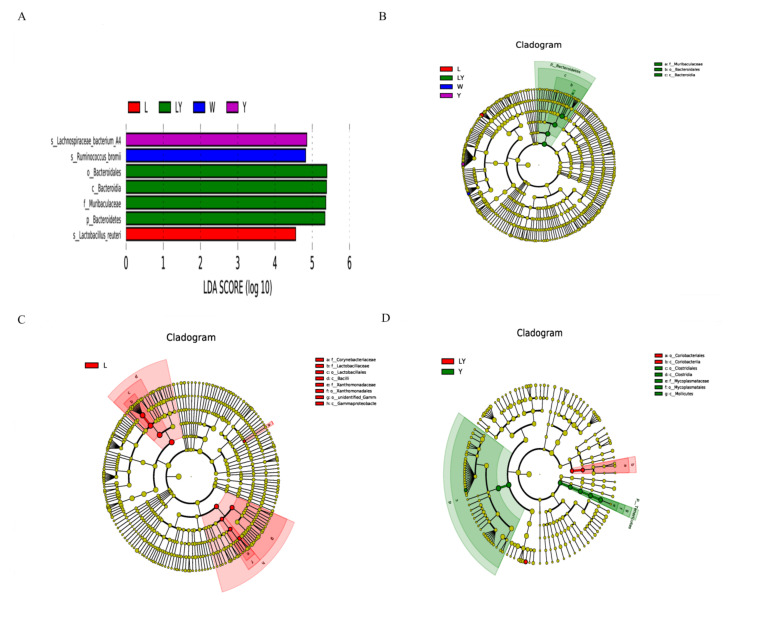
(**A**) Linear discriminant analysis (LDA) discriminant histogram of different experimental groups (L-group, Y-group, LY-group, R-group and W-group), (**B**) LDA Effect Size (LEfSe) multi-level classification tree diagram of different experimental groups (L-group, Y-group, LY-group, R-group and W-group), (**C**) LEfSe multi-level classification tree diagram of L-group and LY-group, (**D**) LEfSe multi-level classification tree diagram of Y-group and LY-group.

Figure 6A clearly shows the microbial species with significant differences among L-group, LY-group, Y-group, and W-group. The dominant species in Y-group, W-group, and L-group were, respectively, *Lachnospiraceae bacterium*, *Ruminococcus bromii*, and *Lactobacillus reuteri*. The dominant species in LY-group included *Bacteroidales*, *Bacteroidia*, *Muribaculaceae,* and *Bacteroidetes*. The microbial species with the significant abundance difference between LY-group and the other four groups were *Bacteroidales*, *Bacteroidia,* and *Muribaculaceae* (Figure 6B). The three dominant genera in gut *Bacteroidales* included *Bacteroides*, *Parabacteroides*, and *Prevotella*. Some species of *Bacteroidales* were highly abundant in the human gut and stable both over the lifetime and even across generations of human due to their ability to utilize polysaccharides [57]. Another study implied that intestinal *Bacteroidales* played important roles in the bioactivities of sulfated polysaccharides [58]. It was worth mentioning that the abundance of *Lactobacillus reuteri* (*L. reuteri*), which is a kind of microorganisms beneficial to the intestinal microecology, in L-group was significantly different from that in the other four groups. *L. reuteri* is one of the widely used probiotics in mammals [59]. *L. reuteri*, as a native resident in gastrointestinal tracts of human and animals, had been verified in animal experiments to be able to reduce insulin resistance, hepatic steatosis, and hyperlipidemia and prevent elevated blood sugar and related metabolic syndrome [60].

Figure 6C shows the comparison results between L-group and LY-group. The dominant microorganisms in L-group included *Corynebacteriaceae*, *Lactobacillaceae*, *Lactobacillales*, *Bacilli*, *Xanthomonadaceae*, *Xanthomonadales*, *unidentified_Gammaproteobacteria*, and *Gammaproteobacteria*. The abundances of these microorganisms in LY-group were not relatively low. Figure 6D shows the comparison results between Y-group and LY-group. The dominant microorganisms in LY-group were *Coriobacteriales* and *Coriobacteriia*, whereas the dominant microorganisms in Y-group were *Clostridiales*, *Clostridia*, *Mycoplasmataceae*, *Mycoplasmatales,* and *Mollicutes*. The results indicated that *L. paracasei* H4-11 and *K. marxianus* L1-1 had different effects on the gut microbiota of mice. These differences also suggested that *L. paracasei* H4-11 and *K. marxianus* L1- 1 might have an interaction mechanism, as confirmed in our previous study [20]. The interaction between the two strains affected the intestinal microecology of the mice. Similarly, Wasilewska et al. also reported that lactic acid bacteria and yeasts had a symbiotic relationship, jointly changed the composition of the gut microbiota of mice, and played an anti-inflammatory and immune role [61].

### 3.6. Correlation between Antioxidant Capacity, Other Parameters, and Gut Microbiota in Mice

**Figure 7:**
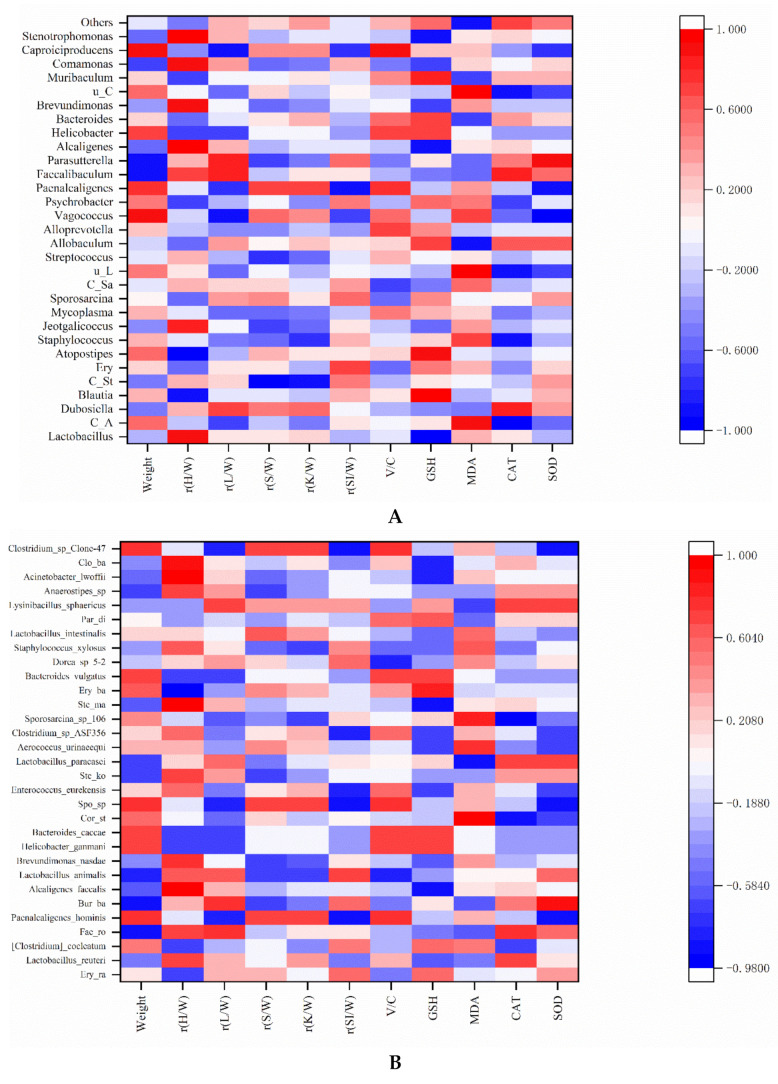
Heatmaps of Spearman’s correlation coefficient of relative abundance microbial composition data (**A**) genus level, (**B**) species level with parameters related to the characteristics of mice’s body characteristics and antioxidant ability of mice’s serum. The full names and abbreviations are as follows, and the abbreviations of the previous nouns are in parentheses. Relative heart weight (r(H/W)), Relative liver weight (r(L/W)), Relative spleen weight (r(S/W)), Relative kidney weight (r(K/W)), Relative small intestine weight (r(SI/W)), Villus height/crypt depth (V/C), Candidatus_Arthromitus (C_A), Candidatus_Stoquefichus (C_St), Erysipelatoclostridium (Ery), Candidatus_Saccharimonas (C_Sa), unidentified_Lachnospiraceae (u_L), unidentified_Corynebacteriaceae (u_C), Erysipelatoclostridium_ramosum (Ery_ra), Faecalibaculum_rodentium (Fae_ro), Burkholderiales_bacterium_YL45 (Bur_ba), Corynebacterium_stationis (Cor_st), Sporosarcina_sp_HW10C2 (Spo_sp), Stenotrophomonas_koreensis (Ste_ko), Stenotrophomonas_maltophilia (Ste_ma), Erysipelotrichaceae_bacterium_I46 (Ery_ba), Parabacteroides_distasonis (Par_di), and Clostridiales_bacterium_CIEAF_022 (Clo_ba).

In this study, we focused the correlation between antioxidant capacity and gut microbiota. Similarly, Lee et al. used the Spearman’s rank order correlations to examine the correlation between another index (vitamin B-12 status) and the levels of antioxidant enzyme activity (SOD, CAT and GPx) and oxidative stress (MDA and Ox-LDL-C) in serum [62]. The correlations between the antioxidant capacity, other parameters (weight, relative organ weight, and villus height/crypt depth) and gut microbiota in the mice of five groups are shown in Figure 7A. At the genus level, *Lactobacillus* and *Blautia* had a significant positive correlation with relative heart weight and *Candidatus_Stoquefichus* had a significant positive correlation with relative kidney weight. There was a significant positive correlation between *Caproiciproducens* and the V/C. It was inferred that *Caproiciproducens* might have a positive effect on the digestion and absorption capacity of mice. Liu et al. demonstrated that the dominant genus of *Caproiciproducens* was a caproic acid-producing bacterium in fermented foods [63]. It was speculated that the production of caproic acid promoted the digestion and absorption capacity of mice. *Atopostipes* was significantly positively correlated with GSH (*r* = 0.9) and *Parasutterella* was significantly positively correlated with SOD (*r* = 0.9). It was inferred that these bacteria might have a certain positive effect on the improvement in antioxidant capacity. Similarity, Li et al. confirmed that *Atopostipes* was involved in the regulation of lipid metabolism and glucose metabolism in serum and liver of mice [64]. Therefore, the antioxidant capacity could be adjusted indirectly by *Atopostipes*. Another study showed that *Parasutterella* could improve GSH and SOD levels by regulating the distribution of gut microbiota of mice [65]. *Lactobacillus* did not show a significant correlation with antioxidant indicators because *Lactobacillus* contains different species. The correlation between *Lactobacillus* and antioxidant indicators required further species analysis.

In addition, *Staphylococcus* and *unidentified_Lachnospiraceae* showed a significant negative correlation with CAT (*r* = −0.9). *Vagococcus* and *Paenalcaligenes* showed a significant negative correlation with SOD (*r* = −0.9). Similarly, *Alcaligenes* and *Stenotrophomonas* were significantly negatively correlated GSH (*r* = −0.9). *Candidatus_Arthromitus* and *unidentified_Corynebacteriaceae* were positively correlated with MDA. At the species level (Figure 7B), *Alcaligenes_faecalis* was significantly negatively correlated with GSH, *Erysipelotrichaceae bacterium* I46 was significantly negatively correlated with GSH. *Corynebacterium_stationis* was positively correlated with MDA and *Stenotrophomonas_maltophilia* was significantly negatively correlated with CAT. The inactivation of the SmeYZ pump of *Stenotrophomonas maltophilia* compromised the virulence-related physiological functions of swimming, flagella formation, oxidative stress susceptibility, biofilm formation, and protease secretion and thus decreased in vivo virulence [66]. This result was consistent with the genus level of the above analysis. It was inferred that these bacteria might have a certain negative effect on the improvement in antioxidant capacity. The effect will be further explored in the future. 

In this study, at the species level (Figure 7B), we found that some beneficial gut microbiota had a positive effect on the antioxidant capacity and other parameters. *Faecalibaculum_rodentium* and *Burkholderiales_bacterium*_YL45 were negatively correlated with body weight and *rysipelotrichaceae_bacterium*_I46 was negatively correlated with the relative heart weight. *Paenalcaligenes_hominis* and *Sporosarcina*_sp_HW10C2 were significantly negatively correlated with relative small intestine weight and SOD, respectively. *Burkholderia* was significantly positively correlated with SOD. Similarly, *Burkholderia cenocepacia* biofilms production had a significant positive correlation with antioxidant capacity (CAT value) [67]. Interestingly, it could be clearly seen that *Lactobacillus paracasei* was positively correlated with GSH (*r* = 0.2052), CAT (*r* = 0.71818), and SOD (*r* = 0.71818), and negatively correlated with MDA (*r* = −0.87208). The results further confirmed that rice-acid fermented with *L. paracasei* H4-11/(*L. paracasei* H4-11 and *K. marxianus* L1-1) might contribute to the enhancement of the antioxidant capacity of mouse serum. Notably, *Lactobacillus* exists in many foods and shows probiotic functions [5,6,7,8,9,10]. Gut microbiota or their metabolites (e.g., lipopolysaccharide) can be used as antigen substances to stimulate intestinal epithelial cells or immune cells and improve host immune function [68]. A recent study discovered that oral probiotics played a role in the intestinal and systemic effects of COVID-19 [69]. Therefore, probiotics may be used as a potential therapy for regulating gut microbiota and improving health. Notably, acidifiers instead of antibacterial drugs are a hot topic in the study on animal diets. The acidifiers in animal diets are well proven to reduce the incidence of diseases in animal intestinal tract, thereby improving the productive performance. Acidifiers in feed help to maintain the optimal intestinal pH for an efficient proteolytic digestive enzyme activity and antimicrobial activity against intestinal pathogens so as to reduce subclinical infection [70]. Fortified fermented rice-acid also belongs to the acidifier group. In this study, the antioxidation status, histological changes and the enhanced gut microbiota provided a new insight into the potential application of rice-acid fermented with *L. paracasei* H4-11/(*L. paracasei* H4-11 and *K. marxianus* L1-1) in animal diets.

## 4. Conclusions

In summary, we explored the positive effects of fortified fermented rice-acid on the antioxidant capacity of mouse serum and gut microbiota. The mice in L-group, LY-group, and R-group kept their weight well and had the better digestion capacity and the mice in the L-group had the better hair quality. Moreover, the inoculation of *L. paracasei* H4-11 and *K. marxianus* L1-1 increased the villus height/crypt depth of mice compared to non-inoculation group (R-group). Notably, the inoculation of *L. paracasei* H4-11 (*L. paracasei* H4-11 and *K. marxianus* L1-1) in fermented rice-acid enhanced the antioxidant capacity of mouse serum. Importantly, *L. paracasei* H4-11 and *K. marxianus* L1-1 might have a synergistic effect on the improvement in the antibacterial function, reduce harmful microorganisms, and increase the beneficial microorganisms in the gut microbiota of the mice. In the future, the hair characteristics of mice and the antibacterial and oxidation mechanisms of the two strains will be further explored. The study gains an insight into the development of fortified fermented rice-acid for regulating the gut microbiota and improving the antioxidant capacity.

## Figures and Tables

**Table 1:** The body weight and the organ coefficient of mice in different experimental groups (L-group, Y-group, LY-group, R-group and W-group).

Group	Weight (g)	Relative Heart Weight (mg/g)	Relative Liver Weight (mg/g)	Relative Spleen Weight (mg/g)	Relative Kidney Weight (mg/g)	Relative Small Intestine Weight (mg/g)
L-group	35.10 ± 0.90 ^b^	5.41 ± 0.56 ^a^	40.53 ± 3.79 ^b^	3.38 ± 0.49 ^a^	16.57 ± 1.67 ^b^	39.05 ± 2.40 ^b^
Y-group	38.81 ± 3.48 ^a^	5.02 ± 0.43 ^b^	38.80 ± 3.25 ^c^	3.10 ± 1.12 ^ab^	16.70 ± 7.05 ^b^	36.61 ± 8.29 ^c^
LY-group	35.11 ± 1.21 ^b^	5.03 ± 0.63 ^b^	41.63 ± 2.97 ^a^	3.19 ± 0.97 ^ab^	16.75 ± 1.43 ^b^	39.96 ± 5.40 ^b^
R-group	35.93 ± 1.74 ^b^	5.08 ± 0.78 ^b^	40.20 ± 3.32 ^b^	3.18 ± 0.49 ^ab^	16.42 ± 1.27 ^b^	41.95 ± 7.06 ^a^
W-group	38.63 ± 2.07 ^a^	5.35 ± 0.61 ^a^	39.21 ± 6.91 ^c^	3.46 ± 0.96 ^a^	18.08 ± 1.78 ^a^	34.03 ± 3.64 ^d^

Results are expressed as the mean ± standard deviation, n = 6. Values with different superscripts in a column indicate significant differences between groups as determined one-way ANOVA followed by the Waller–Duncan multiple range test at *p* < 0.05. ^a, b^ Mean values of Weight, Relative heart weight, and Relative kidney weight within columns with unequal superscript letters were significantly different (*p* < 0.05). ^a–c^ Mean values of Relative liver weight within columns with unequal superscript letters were significantly different (*p* < 0.05). ^a, ab^ Mean values of Relative spleen weight within columns with unequal superscript letters were significantly different (*p* < 0.05). ^a–d^ Mean values of Relative small intestine weight within columns with unequal superscript letters were significantly different (*p* < 0.05).

**Table 2:** The villus height (mm), crypt depth (mm) and the ratio of villus height to crypt depth (V/C) of mice’s intestinal histology in different experimental groups (L-group, Y-group, LY-group, R-group and W-group).

Group	Villus Height (mm)	Crypt Depth (mm)	V/C
L-group	0.483 ± 0.060 ^a^	0.153 ± 0.012 ^a^	3.165 ± 0.405 ^b^
Y-group	0.577 ± 0.039 ^a^	0.150 ± 0.022 ^a^	3.934 ± 0.681 ^a^
LY-group	0.362 ± 0.026 ^bc^	0.120 ± 0.010 ^ab^	3.043 ± 0.406 ^b^
R-group	0.298 ± 0.018 ^c^	0.133 ± 0.014 ^ab^	2.258 ± 0.248 ^c^
W-group	0.422 ± 0.028 ^b^	0.132 ± 0.016 ^ab^	3.270 ± 0.591 ^b^

Results are expressed as the mean ± standard deviation, *n* = 6. Values with different superscripts in a column indicate significant differences between groups as determined by one-way ANOVA followed by a Waller–Duncan multiple range test at *p* < 0.05. ^a–c^ Mean values of Villus height and V/C within columns with unequal superscript letters were significantly different (*p* < 0.05)**.** ^a, ab^ Mean values of Crypt depth within columns with unequal superscript letters were significantly different (*p* < 0.05).

**Table 3:** Analysis of antioxidant ability (glutathione (GSH), malonaldehyde (MDA), catalase (CAT), and superoxide dismutase (SOD)) of mice’s serum in different experimental groups (L-group, Y-group, LY-group, R-group and W-group).

Group	GSH (umol/L)	MDA (mmol/L)	CAT (U/mL)	SOD (U/mL)
L-group	27.132 ± 6.203 ^b^	0.776 ± 0.175 ^b^	11.624 ± 3.111 ^b^	2.138 ± 0.325 ^a^
Y-group	36.735 ± 4.515 ^a^	1.020 ± 0.218 ^ab^	9.743 ± 0.964 ^c^	1.569 ± 0.138 ^b^
LY-group	29.503 ± 6.604 ^b^	0.687 ± 0.125 ^b^	15.644 ± 4.618 ^a^	2.292 ± 0.201 ^a^
R-group	28.135 ± 3.872 ^b^	1.200 ± 0.367 ^a^	9.607 ± 2.323 ^c^	1.591 ± 0.089 ^b^
W-group	13.315 ± 4.829 ^c^	1.127 ± 0.257 ^a^	10.781 ± 3.088 ^b^	1.322 ± 0.152 ^b^

Results are expressed as the mean ± standard deviation, *n* = 6. Values with different superscripts in a column indicate significant differences between groups as determined by one-way ANOVA followed by a Waller–Duncan multiple range test at *p* < 0.05. ^a–c^ Mean values of GSH and CAT within columns with unequal superscript letters were significantly different (*p* < 0.05). ^a, b^ Mean values of MDA and SOD within columns with unequal superscript letters were significantly different (*p* < 0.05).

**Table 4:** Alpha indices statistics of intestinal microbial diversity in different experimental groups (L-group, Y-group, LY-group, R-group and W-group).

Group	Observed Species	Shannon	Simpson	Chao1	ACE	Goods Coverage	PD_Whole_ Tree
L-group	202	3.582	0.723	262.286	288.101	0.980	24.576
Y-group	127	3.510	0.781	197.685	205.261	0.987	15.055
LY-group	108	2.674	0.632	148.113	159.118	0.990	13.465
R-group	131	3.128	0.724	194.636	221.177	0.985	16.435
W-group	179	2.679	0.566	287.020	321.170	0.977	19.783

Results are expressed the as mean.

## Data Availability

The data in this study are available on request from the author.
